# Re: Incidence and treatment of malignant tumors of the genitourinary tract in renal transplant recipients

**DOI:** 10.1590/S1677-5538.IBJU.2018.0406

**Published:** 2019

**Authors:** Michael S. Floyd, Altaf Q Khattak

**Affiliations:** 1 Department of Reconstructive Urology, St Helens & Knowsley Hospital NHS Trust Whiston Hospital, Liverpool, United Kingdom

*To the editor,*

We read with interest the recent paper by Ochoa - López et al. examining the incidence of genitourinary malignancies in renal transplant patients (1). An impressive retrospective analysis of 1256 patients over 48 years is described with a cumulative incidence of 7% for all malignancies identified. Specific to genitourinary neoplasia 16 transplant recipients developed malignancies with 18 separate tumours noted in total. Renal cell carcinoma accounted for 7 with 3 tumours developing in the graft and urothelial carcinoma noted in 6 patients (1). Only 2 developed prostate carcinoma.

Specific to the urothelial carcinoma subgroup, all were non muscle invasive tumours managed with endoscopic resection alone but 1 had nephroureterectomy of the graft performed for upper tract neoplasia.

The authors proceed to discuss risk factors for post - transplant malignancy identifying tobacco use and immunosuppression as predominant ones. Importantly the difficulty in treating transplant patients with bladder cancer is further highlighted due to concerns over intravesical chemotherapy but the authors acknowledge that cystectomy remains an option although no patient in this series underwent radical cystoprostatectomy.

The authors should acknowledge that geographical variations exist in the development of transitional cell carcinoma following transplantation with higher incidences noted in Taiwan (2). A 34 - year study involving 2355 primary transplant patients has previously identified the dysfunction of the antiviral mechanism of the immune system and role of oncogenic viruses in the development of transplant malignancies (3). A separate study has identified Chinese herb use and ingestion of water contaminated with arsenic as specific risk factors for the development of post - transplant transitional cell carcinoma in Taiwan (4).

Shum et al. reported a case of lymphoproliferative disease of the adrenal gland in a transplant recipient treated with rituximab and also a case of bladder cancer treated with radical cystectomy 9 years following cystectomy although the patient succumbed 8 months postoperatively (5).

We reported a case of a 63 year old transplant patient with a triumvirate of genitourinary malignancies occurring 41 years following a live donor transplant (6). The patient was initially thought to have a distal ureteric tumour of the transplanted kidney and was treated with total urinary tract exenteration following which he survived on dialysis for 3 years. Final histological analysis revealed a pT2G3 ureteric tumour, a concomitant pT1G3 bladder tumour and a T2a Gleason six incidental prostate cancer (6).

Chakera et al. have reported a case of renal cell carcinoma of the graft occurring 8 years post transplantation treated with partial nephrectomy and high intensity focussed ultrasound (7).

Weinstein et al. have further reported a case of a 29 year old kidney transplant patient who had undergone conversion of a ureterosigmoidostomy to an ileal conduit and subsequently developed adenocarcinoma of the colon.

Therefore, as the incidence and complexity of genitourinary tumours in transplant recipients increases due to improved immunosuppression and increasing numbers of transplants being performed it is necessary for non - transplant urologists to be aware of the potential uropathological presentations that are unique to this patient cohort.
